# Development of clinical pharmacy services for intensive care units in Korea

**DOI:** 10.1186/2193-1801-3-34

**Published:** 2014-01-17

**Authors:** Jeong Mee Kim, So Jin Park, You Min Sohn, Young Mee Lee, Catherine Seonghee Yang, Hye Sun Gwak, Byung Koo Lee

**Affiliations:** College of Pharmacy & Division of Life and Pharmaceutical Sciences, Ewha Womans University, Seoul, Korea; Department of Pharmacy, Samsung Medical Center, Seoul, Korea

**Keywords:** Clinical pharmacist, Intervention, Intensive care unit

## Abstract

**Objective:**

To be utilized for the development of pharmacists’ intervention service by determining factors which affect pharmacists’ prescription interventions.

**Setting:**

Patients who were admitted to intensive care units (ICUs) in internal medicine departments in Korea.

**Methods:**

Data including age, gender, clinical departments, length of hospital stay, status of organ dysfunction, intervention status, frequently intervened drugs, and health care providers’ questions were prospectively collected in ICUs in the department of internal medicine in a tertiary teaching hospital from January to December, 2012.

**Main outcome measure:**

Primary outcome was factors which affect pharmacists’ prescription interventions. Secondary outcomes included frequencies of the intervention, intervention acceptance rates, intervention issues, and frequently intervened drugs.

**Results:**

A total of 1,213 prescription interventions were made for 445 patients (33.1%) of the 1,344 patients that were analyzed. Length of hospital stay was significantly longer for the group that needed pharmacists’ interventions (p < 0.001). Pharmacists’ intervention requirements were significantly higher in patients with kidney dysfunction (p < 0.001). The percentage of intervention accepted was 96.8%, and interventions that were common were as follows (in order): clinical pharmacokinetic service, dosage or dosing interval changes, dosing time changes or dose changes, and total parenteral nutrition consultation. The five medications with the highest intervened frequency were (in order) vancomycin, famotidine, ranitidine, meropenem, and theophylline.

**Conclusion:**

The need for pharmacists’ prescription interventions was highest among patients with longer length of stay and patients with kidney dysfunction. Based on these findings, prescription intervention activities could be initiated with severely ill patients. The results could be utilized in countries which are planning to develop pharmacists’ intervention service.

## Introduction

Major activities of hospital pharmacists in countries with advanced clinical pharmacies, such as the U.S, can be summarized as ones that provide safe medication administration to patients. In those countries, clinical pharmacists have historically performed prescription interventions in fulfilling their clinical duties as part of health care team. Many studies have reported that such activities contribute greatly patient safety and appropriate medication use bringing about economic benefits (Gillespie et al. 2009; Saokaew et al. 2009; Kopp et al. 2007; Scarsi et al. 2002; Devlin et al. 1997).

In contrast, in Asia and other developing countries, due to the lack of awareness of the role of or the need for clinical pharmacists, clinical activities of hospital pharmacists are not as encouraged as in the U.S. (Khalili et al. 2011; Nissen 2009). This situation is the same in Korea in which clinical pharmacists’ role in hospital pharmacy has a greater emphasis on dispensing and drug distribution and a relatively smaller emphasis on clinical pharmacy interventions.

Activities to improve inpatient services have begun in 2000 in Korea with the separation of dispensing and prescribing functions, which led to the widespread use of aseptic preparations of injectable drugs, the expansion of the use of unit doses, and the more widespread use of daily preparation system. Although it is true that large variations exist among hospitals, major hospitals provide pharmacy services that are comparable to those in countries such as drug information services, clinical pharmacokinetic consultation services, the development of specialized medication counseling, and the operation of services tailored to the needs of specific patients in clinical divisions.

Samsung Medical Center, in which the present study was conducted, is one of the major five hospitals in Korea, consisting of a main hospital and a cancer treatment hospital. The main hospital has 1,900 beds, and the cancer treatment hospital has over 600 inpatient beds, and both hospitals have ICUs. The hospital founded the first ‘Department of Critical Care Medicine’ in Korea in March 2013, which is an independent medical department for ICUs.

**Figure 1:**
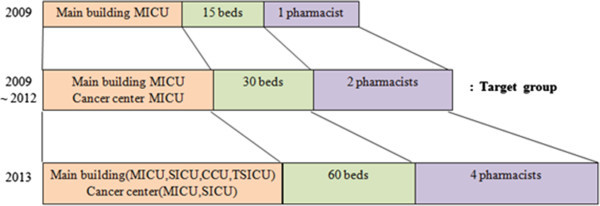
**Intervention service of intensive care unit pharmacists.** MICU: medical intensive care unit, SICU: surgical intensive care unit, CCU: coronary care unit, TSICU: trauma surgical intensive care unit.

The pharmacy in this study began a clinical service for 15 bed Medical ICU unit in February 17, 2009 by creating a team consisting of one clinical pharmacist and the ICU medical staff. Based on a positive feedback on clinical pharmacist’s activities of one year period in 2010, two clinical pharmacists became in charge of a full-scale ICU service in the department of internal medicine in the main hospital and the cancer treatment hospital (a total of 30 beds) (Figure 1). In March, 2013, the department of critical care medicine (CCM) was established. At the request of the CCM department, patients in the departments of general surgery, division of cardiology, thoracic surgery, and general surgery in the cancer treatment hospital were added to the scope of patients receiving such services, and currently, there are a total of four pharmacists in charge of ICU patients. The ICU pharmacists are clinical pharmacists licensed by the U.S. Board of Pharmacy Specialties (BPS) or by the Korean Board of Pharmaceutical Specialties and have more than five years of work experience. They follow the Fundamental, Desirable, Optimal Critical Care Pharmacist Activities Guidelines stated in the Position Paper on Critical Care Pharmacy Services created by American College of Clinical Pharmacy (ACCP) and the Society of Critical Care Medicine (SCCM) (2000).

They perform medication profile management, prescription intervention activity, ADR monitoring, and therapeutic drug monitoring (TDM) work, as well as TPN consultation referrals and participate in the nutrition support team (NST) rounds. In addition, they provide information on new drugs and pharmaceuticals of interest. Other clinical pharmacist activities include providing information on changes in drug supply and medication dispensing as well as providing insurance-related information and performing education for health care professionals.

ICU patients in particular have a high risk and a high frequency of adverse drug reactions (ADR) when prescription errors occur (Leape 1997). This is due to their critical condition, multi-drug use, and the continuing changes in their pharmacokinetic parameters. Thus, a plethora of research has reported that pharmacists’ participation in the pharmacological treatment of ICU patients can lower the frequency of prescription errors or ADRs, and improve treatment outcomes including reduction of length of hospital stay (Saokaew et al. 2009; Kaushal et al. 2008; Kane et al. 2003; Calabrese et al. 2001; Kaboli et al. 2006). However, no particular study on this topic has been conducted in Korea.

### Aim of study

The study aim was to develop pharmacist intervention service by examining factors affecting pharmacists’ prescription interventions, issues with interventions, and the effect of prescription intervention activities, thereby, being utilized for the development of pharmacists’ intervention service.

## Method

The present study was prospectively conducted on patients who were admitted to ICUs in a tertiary teaching hospital in Korea from January to December, 2012. Data collected were age, gender, Simplified Acute Physiology Score (SAPS) III, clinical departments, length of hospital stay, type of organ dysfunction (kidney or liver dysfunction), intervention status, and frequently intervened drugs. All prescriptions were checked for drug choice appropriateness, dose and dose reductions according to organ dysfunctions, administration routes, compatibilities, indications, interactions, and adverse drug reactions. Renal dysfunction was defined as the creatinine clearance less than 50 mL/min. Hepatic dysfunction was defined as the aspartate aminotransferase or alanine aminotransferase ≥ normal value × 3. For the primary outcomes, factors affecting pharmacist interventions were evaluated. With respect to the secondary outcomes, the followings were obtained: the number of interventions, intervention acceptance rates, intervention issues, frequently intervened drugs.

Continuous variables were compared by the Student’s *t*-test. If the variables were not normally distributed, as determined by the one-sample Kolmogorov-Smirnov, additional Mann–Whitney tests were used. The Chi-square or Fisher’s exact test was used to compare categorical variables. A multiple regression model was used to investigate the factors that independently affected the pharmacist intervention. The data were analyzed using Statistical Package for Social Sciences Version 12.0 for Windows (SPSS INC., Chicago, IL, USA). A P-value of less than 0.05 was considered statistically significant.

### Ethical approval

This study was conducted with the approval of the institutional review board (IRB) of Samsung Medical Center (IRB # 2012-07-117).

## Results

**Figure 2:**
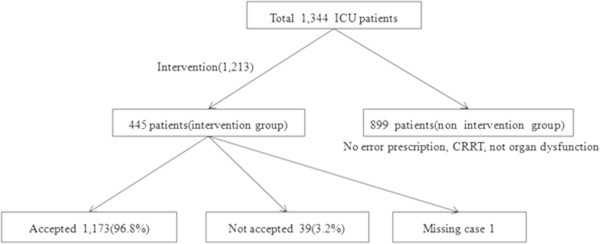
**Study profile of clinical pharmacist interventions.**

During the study period, the number of patients who were admitted to the ICU at the department of internal medicine was 1,344, and the number of patients who needed pharmacists’ interventions was 445 (33.1%). A total of 1,213 interventions were performed for the 445 patients (Figure 2). The average values of SAPS III was 55.7 in the main hospital patients and 62.8 in the cancer treatment hospital patients.

As shown in Table 1, there was no significant difference in age and sex between patients with and without pharmacist interventions. The relationship between the type of clinical departments and the number of pharmacist interventions was significant (p < 0.001), with the division of gastroenterology having a lower rate of patients in need of intervention than other divisions. In contrast, the divisions of pulmonary and hematology/oncology, showed a higher rate of patients in need of intervention compared to the other departments. In the length of hospital stay, the group in need of clinical pharmacist interventions had significantly longer hospital stay than the other group (p < 0.001). The need for intervention was influenced by the presence of kidney dysfunction (p < 0.001), so patients with kidney dysfunction required more clinical pharmacist intervention than patients without kidney dysfunction (Table 1).

**Table 1:** **Patient characteristics according to the presence and absence of pharmacist intervention requirements**

		Total (n = 1,344)	Required pharmacist intervention		***P***-value
			Yes (n = 445)	No (n = 899)	
Age, median (range), y		64.0 (10–95)	64.0 (18–94)	64.0 (10–95)	0.648
Sex, n (%)					0.367
	Male	856	291 (65.4)	565 (62.8)	
	Female	488	154 (34.6)	334 (37.2)	
Clinical departments, n (%)					<0.001
	Gastroenterology	139	19 (4.3)	120 (13.3)	
	Pulmonary	423	164 (36.9)	259 (28.8)	
	Nephrology	84	19 (4.3)	65 (7.2)	
	Hematology & Oncology	521	189 (42.5)	332 (36.9)	
	Infectious Diseases	98	31 (7.0)	67 (7.5)	
	Others	79	23 (5.2)	56 (6.2)	
Length of hospital stay, median (range), day^1^		4.0 (1–195)	9.0 (1–195)	2.0 (1–109)	<0.001
Clinical conditions, n (%)					
	Renal dysfunction				<0.001
	Yes	419	173 (38.9)	246 (27.4)	
	No	925	272 (61.1)	653 (72.6)	
	Hepatic dysfunction				0.062
	Yes	225	62 (13.9)	163 (18.1)	
	No	1,119	383 (86.1)	736 (81.9)	

^1^Two patients who were staying in hospital at the point of data collection were excluded.

The results of the multivariable analysis on the variables whose significance level was less than 0.1 in Table 1, except for the clinical departments due to the possible multicolinearity problem, showed that the odds of belonging to the intervention group increased 1.160 times with a 1 day increase in the length of hospital stay (95% CI, 1.134–1.186). The significantly higher likelihood (OR 1.759, 95% CI, 1.340–2.309) to need pharmacist interventions was found in patients with kidney dysfunction (Table 2).

**Table 2:** **Factors associated with pharmacist interventions**

	OR (95% CI)^a^	***P***-value
Length of hospital stay, day	1.160 (1.134–1.186)	<0.001
Renal dysfunction		
No	1	-
Yes	1.759 (1.340–2.309)	<0.001
Hepatic dysfunction		
No	1	-
Yes	0.918 (0.639–1.318)	0.642

OR, odds ratio; CI, confidence interval.

^a^Adjusted for length of hospital stay, renal dysfunction, and hepatic dysfunction.

A total of 1,213 interventions were made for 445 patients among the 1,344 participating patients who were admitted to ICUs during the one year study period, and the frequency was the highest for patients with one intervention (47.4%). As for the intervention acceptance rate, in a total of 1,212 interventions except for one missing case out of 1,213, 1,173 interventions (96.8%) were accepted. The results of intervention issues showed that significant differences existed in acceptance rates depending on intervention issues (p = 0.001). Table 3 showed that the acceptance rate for interventions for medication change was 80.0%, which was lower than the average acceptance rate of 96.8%. The intervention issue with the highest frequency was clinical pharmacokinetic service with 286 interventions, followed by dosage decrement/dosing interval increment with 243 interventions, dosing time or dose with 212 interventions, and TPN consultation with 114 interventions. Thus, the issues related to drug dose (dose, dosing interval, and dosing time).

**Table 3:** **Types of intervention issues and acceptance rates**

Intervention issue		Total (n = 1,212)	Acceptance of Intervention	
			Yes (n = 1,173)	No (n = 39)
Clinical pharmacokinetic service		286 (100.0)	282 (98.6)	4 (1.4)
Total parenteral nutrition		114 (100.0)	107 (93.9)	7 (6.1)
Dosing	Dosage decrement or dosing interval increment	243 (100.0)	232 (95.5)	11 (4.5)
	Dosing time or dose	212 (100.0)	208 (98.1)	4 (1.9)
	Dosage increment or dosing interval decrement	92 (100.0)	90 (97.8)	2 (2.2)
	Dosing route	19 (100.0)	19 (100.0)	0 (0.0)
Medication change	Medication form	47 (100.0)	45 (95.7)	2 (4.3)
	Discontinuation of medication	20 (100.0)	19 (95.0)	1 (5.0)
	Medication change	20 (100.0)	16 (80.0)	4 (20.0)
	New drug recommendation	16 (100.0)	16 (100.0)	0 (0.0)
Adverse drug reaction		79 (100.0)	78 (98.7)	1 (1.3)
Medical insurance		17 (100.0)	17 (100.0)	0 (0.0)
Fluid compatibility		15 (100.0)	13 (86.7)	2 (13.3)
Drug interaction		13 (100.0)	12 (92.3)	1 (7.7)
Others		19 (100.0)	19 (100.0)^1^	0 (0.0)
Total		1,212 (100.0)	1,173 (96.8)	39 (3.2)

Values are expressed as the number (percentage).

^1^Composed of drug omission 10 cases, prescription check 6 cases, and close monitoring recommendation 3 cases.

*P*-value = 0.001.

Table 4 shows intervention issues for the top five drugs (vancomycin, famotidine, ranitidine, meropenem, theophylline) with the highest number of interventions, indicating significantly different intervention issues depending on the drug. For vancomycin and theophylline, the clinical pharmacokinetic service was the most intervened issues. On the contrary, for famotidine, ranitidine, and meropenem, the rates of dosage decreases and dosing interval increases were higher than 50%. The overall frequencies of prescriptions of vancomycin, famotidine, ranitidine, meropenem, and theophylline were 1467, 1018, 1105, 1438, and 403, respectively.

**Table 4:** **Intervention issues of top five medications**

Medication	Vancomycin	Famotidine	Ranitidine	Meropenem	Theophylline
Intervention Issue					
Clinical pharmacokinetic service	201 (85.2)	0 (0.0)	0 (0.0)	0 (0.0)	20 (47.6)
Dosage increment or dosing interval decrement	5 (2.1)	4 (6.5)	2 (3.4)	13 (22.8)	5 (11.9)
Dosage decrement or dosing interval increment	8 (3.4)	42 (67.7)	36 (62.1)	32 (56.1)	1 (2.4)
Medication form	1 (0.4)	0 (0.0)	0 (0.0)	0 (0.0)	1 (2.4)
Dosing time or dose	19 (8.1)	4 (6.5)	5 (8.6)	8 (14.0)	10 (23.8)
Dosing route	1 (0.4)	3 (4.8)	1 (1.7)	0 (0.0)	1 (2.4)
Discontinuation of medication	0 (0.0)	3 (4.8)	2 (3.4)	0 (0.0)	1 (2.4)
Medication change	0 (0.0)	1 (1.6)	0 (0.0)	0 (0.0)	0 (0.0)
Adverse drug reaction	1 (0.4)	5 (8.1)	11 (19.0)	2 (3.5)	1 (2.4)
Fluid compatibility	0 (0.0)	0 (0.0)	0 (0.0)	1 (1.8)	0 (0.0)
Drug interaction	0 (0.0)	0 (0.0)	0 (0.0)	1 (1.8)	1 (2.4)
Others	0 (0.0)	0 (0.0)	1 (1.7)^1^	0 (0.0)	1 (2.4)
Total	236 (100.0)	62 (100.0)	58 (100.0)	57 (100.0)	42 (100.0)

Values are expressed as the number (percentage).

^1^Drug Omission.

*P*-value < 0.001.

## Discussion

The intervention was conducted to 445 patients out of 1,344 study populations, which was 33.1%. Other studies showed various intervention rates; one study showed 12 out of 50 ICU patients whereas another study reported much higher intervention rate of 47% (Devlin et al. 1997; Khalili et al. 2013).

Cases that did not need interventions were as follows: first, cases without an error in the doctor’s prescription; second, temporary admissions to ICUs for continuous renal replacement therapy; and third, cases that did not need additional dose adjustment such as patients without organ dysfunction. The third group accounted for a significant portion of no intervention requirement, therefore, the cases in which patients had organ dysfunction such as kidney dysfunction became mostly the target of intervention.

The result of the relationship between the number of interventions and the type of clinical departments was significant (p < 0.001), with the division of hematology & oncology and pulmonary having the highest intervention rate, and the division of gastroenterology having a lower rate of patients. Most patients with a respiratory disease had diseases such as sepsis, pneumonia, and acute respiratory distress syndrome, which are conditions that usually require medications that need dose adjustment such as antibiotics, sedatives, analgesics, and steroids. On the other hand, the division of gastroenterology did not need many pharmacist interventions, although there are a large number of patients with liver disease. In the case of patients with acute liver disease in ICU, the low rate of pharmacist intervention are due to relatively low number of medications administered for treatment, fewer dose adjustment guidelines for liver dysfunction compared with kidney dysfunction, and the already established protocols in the clinical department with respect to drug treatment.

In clinical conditions, the percentage of patients with renal dysfunction was higher among patients requiring pharmacist’s interventions compared to those without pharmacist’s intervention requirement. Therefore, kidney function is considered to be correlated directly with greater pharmacists’ interventions.

There were 246 patients with renal failure in the group without intervention although the portion was significantly lower than in the group with intervention. The main reason that a significant portion of no intervention requirement was the cases in which patients had kidney dysfunction was because adjusted drug dosing was initiated to patients with severely impaired renal function.

In Kane et al.’s review, the issues that require additional attention included dose adjustment for decreased kidney and liver function, prevention and monitoring of ADRs, drug interactions caused by complex drug regimens, nutritional assessment due to poor oral intake and change in calorie needs, compatibility checks due to a patient’s extensive list of intravenous medications, and treatment and prevention of life-threatening infections, which are the same as the intervention issues encountered in the present study (Kane et al. 2003).

Hospital stay was found to be significantly longer for the group needing pharmacist interventions (p < 0.01). It can be interpreted that patients with longer hospital stay are more likely to need pharmacist’ proactive intervention because of the higher probability of severe illness among these patients, with many complications or delayed treatment in this group.

As for the acceptance rate of intervention, among the total of 1,212 evaluated interventions excluding 1 missing case, 1,173 cases were accepted (96.8%). This result was comparable to the 96.5% acceptance rate in the study by Vessal et al. (Vessal 2010). The main reason for the non-acceptance was prescription change or cancellation due to the alteration of patient health status.

Many studies reported that clinical pharmacists’ activities in ICUs have a positive effect in the patient safety, as illustrated by Calabrese et al. who reported that medication error was reduced by 3.3% with routine activities of clinical pharmacists in ICUs. Other studies showed that 25% of inappropriate drug concentrations were prevented by therapeutic drug monitoring, ADE decreased by 66%, and a potential estimated annual cost saving of 270,000 US dollars (Kane et al. 2003; Calabrese et al. 2001; Dager and Alberson 1992; Leape et al. 1999).

Although this study is not sufficient to prove the effectiveness of clinical pharmacists’ activities in ICUs, pharmacists in charge of ICU patients have already taken on a role as an important member of the health care team in ICUs. As a result, the number of dedicated pharmacist doubled; these personnel are subsequently actively providing drug education to other health care professionals based on the results of the intervention activities during the period, and actively participating in the development of standard clinical pathways.

Medicine is a rapidly growing field. However, the traditional way of practicing medicine places too much burden on doctors, preventing them from coping with the rapidly developing environment as well as threatening patient safety (Leape et al. 1999). Therefore, in developed countries, team-based treatment is becoming more common and professionals in different fields, such as pharmacists, nurses, and nutritionists, are cooperating with each other, creating synergy as well as increasing collaboration among clinical departments, which continuously highlights the importance of multidisciplinary treatment. According to Peter et al., clinical pharmacists are uniquely trained in therapeutics and provide comprehensive drug management of patients and provide education about drug management to other health care providers (Kaboli et al. 2006). Pharmacist intervention outcomes include pharmacoeconomics, health-related quality of life, patient satisfaction, medication appropriateness, and adverse drug events. Therefore, to achieve complete medication safety, pharmacists’ participation in the treatment setting is essential, and if not permitted by circumstances, at the very least, patients with relatively severe illnesses should have the opportunity to receive a wide range of prescription intervention activities by clinical pharmacists. This is why pharmacists should be present in the clinical setting in the current environment to achieve optimum improvement in the quality of drug treatment with overall quality of care.

## Conclusion

The need for pharmacists’ prescription interventions was high in the groups with long hospital stays and kidney dysfunction among the ICU patients. The acceptance rate of pharmacist’ prescription interventions was 96.8%, and the intervention issue with the highest frequency was related to drug dosage. Drugs with a high frequency of intervention were antibiotics and antiulcer drugs. Based on these results, we suggest that pharmacist’ prescription intervention should be started with patients who have relatively high disease severity. The results could be utilized in countries which are planning to develop pharmacists’ intervention service.

### Impact statements

- There is a positive impact on developing clinical pharmacy services in intensive care unit.
- These practices may lead to a step-up in terms of the quality of drug treatment in Korean hospitals.
